# *N*-(4,6-Dimethyl­pyrimidin-2-yl)-1,3-benzothia­zol-2-amine

**DOI:** 10.1107/S1600536811044631

**Published:** 2011-10-29

**Authors:** Shaaban K. Mohamed, Peter N. Horton, Mahmoud A. A. El-Remaily, Hussam Abdel-Ghany, Seik Weng Ng

**Affiliations:** a Chemistry and Environmental Division, Manchester Metropolitan University, Manchester M15 6BH, England; b School of Chemistry, University of Southampton, Southampton SO17 1BJ, England; c Department of Chemistry, Faculty of Science, Sohag University, Egypt; d Department of Chemistry, University of Malaya, 50603 Kuala Lumpur, Malaysia; e Chemistry Department, King Abdulaziz University, PO Box 80203 Jeddah, Saudi Arabia

## Abstract

In the title compound, C_13_H_12_N_4_S, an amino N atom is connected to a 1,3-benzothia­zole fused-ring system and a dimethyl-substituted pyrimidine ring, these components being aligned [inter­planar dihedral angle = 1.9 (1)°]. The secondary amino N atom forms an inter­molecular N—H⋯N hydrogen bond to an N atom of the fused ring of an adjacent mol­ecule, generating a centrosymmetric cyclic hydrogen-bonded dimer [graph set *R*_2_^2^(8)].

## Related literature

For the structure of *N*-(4,6-dimethyl­pyrimidin-2-yl)-1*H*-benzimidazol-2-amine, see: Mohamed *et al.* (2011 ▶). For graph-set analysis, see: Etter *et al.* (1990 ▶).
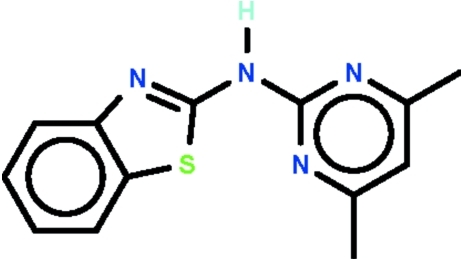


## Experimental

### 

#### Crystal data

C_13_H_12_N_4_S
*M* *_r_* = 256.34
Monoclinic, 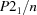

*a* = 6.7608 (2) Å
*b* = 8.5154 (2) Å
*c* = 20.6503 (9) Å
β = 97.237 (2)°
*V* = 1179.39 (7) Å^3^
*Z* = 4
Mo *K*α radiation
μ = 0.26 mm^−1^
*T* = 120 K
0.24 × 0.14 × 0.08 mm

#### Data collection

Bruker–Nonius Roper CCD camera on κ-goniostat diffractometer
Absorption correction: multi-scan (*SADABS*; Sheldrick, 1996 ▶) *T*_min_ = 0.940, *T*_max_ = 0.980
11943 measured reflections
2704 independent reflections
2100 reflections with *I* > 2σ(*I*)
*R*_int_ = 0.061

#### Refinement

*R*[*F* ^2^ > 2σ(*F* ^2^)] = 0.048
*wR*(*F* ^2^) = 0.126
*S* = 1.02
2704 reflections
169 parameters
H atoms treated by a mixture of independent and constrained refinement
Δρ_max_ = 0.29 e Å^−3^
Δρ_min_ = −0.46 e Å^−3^

### 

Data collection: *COLLECT* (Hooft, 1998 ▶); cell refinement: *DENZO* (Otwinowski & Minor, 1997 ▶) and *COLLECT*; data reduction: *DENZO* and *COLLECT*; program(s) used to solve structure: *SHELXS97* (Sheldrick, 2008 ▶); program(s) used to refine structure: *SHELXL97* (Sheldrick, 2008 ▶); molecular graphics: *X-SEED* (Barbour, 2001 ▶); software used to prepare material for publication: *publCIF* (Westrip, 2010 ▶).

## Supplementary Material

Crystal structure: contains datablock(s) global, I. DOI: 10.1107/S1600536811044631/zs2156sup1.cif

Structure factors: contains datablock(s) I. DOI: 10.1107/S1600536811044631/zs2156Isup2.hkl

Supplementary material file. DOI: 10.1107/S1600536811044631/zs2156Isup3.cml

Additional supplementary materials:  crystallographic information; 3D view; checkCIF report

## Figures and Tables

**Table 1:** Hydrogen-bond geometry (Å, °)

*D*—H⋯*A*	*D*—H	H⋯*A*	*D*⋯*A*	*D*—H⋯*A*
N2—H2⋯N1^i^	0.89 (3)	2.27 (3)	3.142 (2)	168 (2)

Symmetry code: (i) 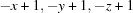.

**Fig. 1.**
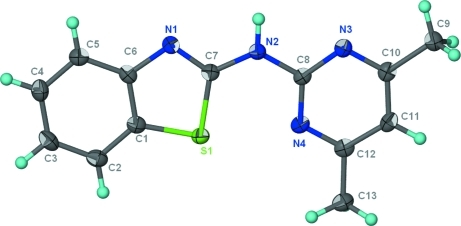
Thermal ellipsoid plot (Barbour, 2001) of C13H12N4S at the 70% probability level. Hydrogen atoms are drawn as spheres of arbitrary radius.

## supplementary crystallographic information

### Comment

In an earlier study, we reported the structure of N-(4,6-dimethylpyrimidin-2-yl)-1H-benzimidazol-2-amine (Mohamed et al., 2011). The benzimidazole portion of that molecule was replaced by a benzothiazole unit in the present study, giving the title compound C_13_H_12_N_4_S (Scheme I). In this molecule, an amino N atom is connected to a benzothiazole fused-ring system and a dimethyl-substituted pyrimidine ring, these being aligned [inter-ring dihedral angle, 1.9 (1)°] (Fig. 1). The amino N atom forms an intermolecular N—H···N hydrogen bond to the N atom of the fused-ring of an adjacent molecule (Table 1) to generate a centrosymmetric cyclic hydrogen-bonded dimer [graph set R</i<>^2^_2_(8) (Etter et al., 1990)].

### Experimental

2-(1,3-Benzothiazol-2-yl)guanidine (0.05 mol) was heated in acetylacetone solution (0.10 mol, approx. 10 ml) in the presence of a few drops of acetic acid at 473 K for 1 h. The mixture was cooled and the product was collected and recrystalized from ethanol to give the title compound (m.p. 513 K) in 85% yield.

### Refinement

Carbon-bound H-atoms were placed in calculated positions (C—H, 0.95 to 0.98 Å) and were included in the refinement in the riding model approximation, with *U*_iso_(H) set to 1.2 to 1.5*U*_eq_(C). The amino H-atom was located in a difference Fourier map, and was freely refined. The reflections (-1 2 3) and (0 1 2) were omitted because to bad agreement.

### Crystal data

**Table:**

C_13_H_12_N_4_S	*F*(000) = 536
*M**_r_* = 256.34	*D*_x_ = 1.444 Mg m^−^^3^
Monoclinic, *P*2_1_/*n*	Melting point: 513 K
Hall symbol: -P 2yn	Mo *K*α radiation, λ = 0.71073 Å
*a* = 6.7608 (2) Å	Cell parameters from 2630 reflections
*b* = 8.5154 (2) Å	θ = 2.9–27.5°
*c* = 20.6503 (9) Å	µ = 0.26 mm^−^^1^
β = 97.237 (2)°	*T* = 120 K
*V* = 1179.39 (7) Å^3^	Block, colorless
*Z* = 4	0.24 × 0.14 × 0.08 mm

### Data collection

**Table:**

Bruker–Nonius Roper CCD camera on κ-goniostat diffractometer	2704 independent reflections
Radiation source: Bruker–Nonius FR591 rotating anode	2100 reflections with *I* > 2σ(*I*)
graphite	*R*_int_ = 0.061
Detector resolution: 9.091 pixels mm^-1^	θ_max_ = 27.6°, θ_min_ = 3.1°
φ and ω scans	*h* = −8→8
Absorption correction: multi-scan (*SADABS*; Sheldrick, 1996)	*k* = −11→10
*T*_min_ = 0.940, *T*_max_ = 0.980	*l* = −25→26
11943 measured reflections	

### Refinement

**Table:**

Refinement on *F*^2^	Primary atom site location: structure-invariant direct methods
Least-squares matrix: full	Secondary atom site location: difference Fourier map
*R*[*F*^2^ > 2σ(*F*^2^)] = 0.048	Hydrogen site location: inferred from neighbouring sites
*wR*(*F*^2^) = 0.126	H atoms treated by a mixture of independent and constrained refinement
*S* = 1.02	*w* = 1/[σ^2^(*F*_o_^2^) + (0.0525*P*)^2^ + 0.7927*P*] where *P* = (*F*_o_^2^ + 2*F*_c_^2^)/3
2704 reflections	(Δ/σ)_max_ = 0.001
169 parameters	Δρ_max_ = 0.29 e Å^−^^3^
0 restraints	Δρ_min_ = −0.46 e Å^−^^3^

### Fractional atomic coordinates and isotropic or equivalent isotropic displacement parameters (Å^2^)

**Table:**

	*x*	*y*	*z*	*U*_iso_*/*U*_eq_	
S1	0.47670 (7)	0.76071 (6)	0.65817 (3)	0.01754 (17)	
N1	0.6118 (2)	0.55355 (17)	0.58054 (8)	0.0158 (4)	
N2	0.2981 (2)	0.64918 (19)	0.53944 (9)	0.0170 (4)	
H2	0.305 (4)	0.586 (3)	0.5054 (13)	0.034 (7)*	
N3	−0.0130 (2)	0.71820 (18)	0.48927 (8)	0.0165 (4)	
N4	0.1226 (2)	0.83658 (18)	0.59065 (8)	0.0167 (4)	
C1	0.7100 (3)	0.6766 (2)	0.68212 (10)	0.0167 (4)	
C2	0.8414 (3)	0.7057 (2)	0.73865 (10)	0.0203 (5)	
H2A	0.8097	0.7796	0.7702	0.024*	
C3	1.0194 (3)	0.6232 (2)	0.74717 (10)	0.0226 (5)	
H3	1.1108	0.6402	0.7854	0.027*	
C4	1.0668 (3)	0.5154 (2)	0.70042 (10)	0.0215 (5)	
H4	1.1898	0.4602	0.7075	0.026*	
C5	0.9374 (3)	0.4875 (2)	0.64396 (10)	0.0184 (4)	
H5	0.9708	0.4147	0.6122	0.022*	
C6	0.7561 (3)	0.5692 (2)	0.63488 (10)	0.0160 (4)	
C7	0.4608 (3)	0.6453 (2)	0.58687 (10)	0.0152 (4)	
C8	0.1274 (3)	0.7392 (2)	0.54049 (10)	0.0150 (4)	
C9	−0.3381 (3)	0.7869 (2)	0.43244 (11)	0.0201 (4)	
H9A	−0.2945	0.7105	0.4017	0.030*	
H9B	−0.3627	0.8884	0.4105	0.030*	
H9C	−0.4611	0.7497	0.4479	0.030*	
C10	−0.1786 (3)	0.8053 (2)	0.48952 (10)	0.0168 (4)	
C11	−0.2001 (3)	0.9088 (2)	0.54009 (10)	0.0181 (4)	
H11	−0.3180	0.9694	0.5400	0.022*	
C12	−0.0455 (3)	0.9217 (2)	0.59088 (10)	0.0168 (4)	
C13	−0.0551 (3)	1.0316 (2)	0.64689 (11)	0.0217 (5)	
H13A	0.0326	0.9934	0.6851	0.032*	
H13B	−0.1924	1.0371	0.6573	0.032*	
H13C	−0.0117	1.1364	0.6351	0.032*	

### Atomic displacement parameters (Å^2^)

**Table:**

	*U*^11^	*U*^22^	*U*^33^	*U*^12^	*U*^13^	*U*^23^
S1	0.0175 (3)	0.0186 (3)	0.0166 (3)	0.00010 (19)	0.0027 (2)	−0.00291 (19)
N1	0.0172 (8)	0.0131 (8)	0.0166 (9)	−0.0006 (6)	0.0008 (7)	0.0007 (6)
N2	0.0170 (9)	0.0173 (8)	0.0163 (9)	0.0018 (7)	0.0008 (7)	−0.0029 (7)
N3	0.0158 (8)	0.0159 (8)	0.0181 (9)	−0.0013 (6)	0.0033 (7)	0.0016 (7)
N4	0.0183 (8)	0.0145 (8)	0.0181 (9)	−0.0003 (6)	0.0051 (7)	0.0009 (7)
C1	0.0189 (10)	0.0165 (9)	0.0153 (10)	−0.0022 (8)	0.0040 (8)	0.0017 (8)
C2	0.0241 (11)	0.0222 (10)	0.0152 (11)	−0.0031 (8)	0.0048 (9)	−0.0002 (8)
C3	0.0240 (11)	0.0272 (11)	0.0153 (11)	−0.0037 (9)	−0.0021 (9)	0.0023 (8)
C4	0.0188 (10)	0.0229 (10)	0.0221 (12)	0.0016 (8)	−0.0002 (9)	0.0044 (9)
C5	0.0212 (10)	0.0164 (9)	0.0180 (11)	−0.0012 (8)	0.0038 (8)	0.0005 (8)
C6	0.0179 (10)	0.0145 (9)	0.0153 (10)	−0.0035 (7)	0.0011 (8)	0.0022 (8)
C7	0.0168 (10)	0.0131 (9)	0.0160 (10)	−0.0025 (7)	0.0027 (8)	−0.0001 (7)
C8	0.0160 (9)	0.0129 (9)	0.0167 (11)	−0.0014 (7)	0.0047 (8)	0.0014 (7)
C9	0.0189 (10)	0.0208 (10)	0.0203 (11)	−0.0001 (8)	0.0013 (9)	0.0012 (8)
C10	0.0175 (9)	0.0133 (9)	0.0201 (11)	−0.0017 (7)	0.0044 (8)	0.0042 (8)
C11	0.0164 (10)	0.0166 (9)	0.0222 (11)	0.0021 (8)	0.0060 (8)	0.0039 (8)
C12	0.0199 (10)	0.0131 (9)	0.0184 (11)	−0.0033 (7)	0.0065 (8)	0.0028 (8)
C13	0.0233 (11)	0.0188 (10)	0.0238 (12)	−0.0001 (8)	0.0068 (9)	−0.0030 (8)

### Geometric parameters (Å, °)

**Table:**

S1—C1	1.746 (2)	C3—H3	0.9500
S1—C7	1.762 (2)	C4—C5	1.387 (3)
N1—C7	1.305 (2)	C4—H4	0.9500
N1—C6	1.397 (2)	C5—C6	1.401 (3)
N2—C7	1.378 (2)	C5—H5	0.9500
N2—C8	1.388 (2)	C9—C10	1.502 (3)
N2—H2	0.89 (3)	C9—H9A	0.9800
N3—C8	1.342 (3)	C9—H9B	0.9800
N3—C10	1.343 (2)	C9—H9C	0.9800
N4—C8	1.330 (3)	C10—C11	1.388 (3)
N4—C12	1.349 (3)	C11—C12	1.389 (3)
C1—C6	1.400 (3)	C11—H11	0.9500
C1—C2	1.397 (3)	C12—C13	1.496 (3)
C2—C3	1.385 (3)	C13—H13A	0.9800
C2—H2A	0.9500	C13—H13B	0.9800
C3—C4	1.399 (3)	C13—H13C	0.9800
			
C1—S1—C7	88.04 (9)	N1—C7—S1	116.81 (14)
C7—N1—C6	109.77 (16)	N2—C7—S1	122.68 (15)
C7—N2—C8	126.35 (18)	N4—C8—N3	127.80 (18)
C7—N2—H2	115.6 (16)	N4—C8—N2	117.28 (17)
C8—N2—H2	118.1 (16)	N3—C8—N2	114.91 (17)
C8—N3—C10	115.50 (17)	C10—C9—H9A	109.5
C8—N4—C12	116.16 (17)	C10—C9—H9B	109.5
C6—C1—C2	121.59 (18)	H9A—C9—H9B	109.5
C6—C1—S1	110.06 (15)	C10—C9—H9C	109.5
C2—C1—S1	128.35 (16)	H9A—C9—H9C	109.5
C3—C2—C1	117.85 (19)	H9B—C9—H9C	109.5
C3—C2—H2A	121.1	N3—C10—C11	121.32 (18)
C1—C2—H2A	121.1	N3—C10—C9	117.08 (18)
C2—C3—C4	121.07 (19)	C11—C10—C9	121.60 (18)
C2—C3—H3	119.5	C10—C11—C12	118.60 (18)
C4—C3—H3	119.5	C10—C11—H11	120.7
C5—C4—C3	121.16 (19)	C12—C11—H11	120.7
C5—C4—H4	119.4	N4—C12—C11	120.60 (18)
C3—C4—H4	119.4	N4—C12—C13	117.25 (18)
C4—C5—C6	118.41 (19)	C11—C12—C13	122.14 (18)
C4—C5—H5	120.8	C12—C13—H13A	109.5
C6—C5—H5	120.8	C12—C13—H13B	109.5
C1—C6—N1	115.32 (17)	H13A—C13—H13B	109.5
C1—C6—C5	119.92 (18)	C12—C13—H13C	109.5
N1—C6—C5	124.76 (18)	H13A—C13—H13C	109.5
N1—C7—N2	120.51 (18)	H13B—C13—H13C	109.5
			
C7—S1—C1—C6	−0.08 (15)	C8—N2—C7—S1	−0.8 (3)
C7—S1—C1—C2	179.4 (2)	C1—S1—C7—N1	−0.22 (16)
C6—C1—C2—C3	−0.8 (3)	C1—S1—C7—N2	179.59 (17)
S1—C1—C2—C3	179.75 (16)	C12—N4—C8—N3	1.7 (3)
C1—C2—C3—C4	0.6 (3)	C12—N4—C8—N2	−179.41 (16)
C2—C3—C4—C5	0.1 (3)	C10—N3—C8—N4	−0.8 (3)
C3—C4—C5—C6	−0.5 (3)	C10—N3—C8—N2	−179.65 (16)
C2—C1—C6—N1	−179.22 (17)	C7—N2—C8—N4	3.1 (3)
S1—C1—C6—N1	0.3 (2)	C7—N2—C8—N3	−177.90 (17)
C2—C1—C6—C5	0.4 (3)	C8—N3—C10—C11	−0.4 (3)
S1—C1—C6—C5	179.93 (15)	C8—N3—C10—C9	179.09 (17)
C7—N1—C6—C1	−0.5 (2)	N3—C10—C11—C12	0.4 (3)
C7—N1—C6—C5	179.94 (18)	C9—C10—C11—C12	−179.04 (18)
C4—C5—C6—C1	0.3 (3)	C8—N4—C12—C11	−1.6 (3)
C4—C5—C6—N1	179.82 (18)	C8—N4—C12—C13	179.75 (17)
C6—N1—C7—N2	−179.37 (17)	C10—C11—C12—N4	0.6 (3)
C6—N1—C7—S1	0.4 (2)	C10—C11—C12—C13	179.24 (18)
C8—N2—C7—N1	179.05 (17)		

### Hydrogen-bond geometry (Å, °)

**Table:**

*D*—H···*A*	*D*—H	H···*A*	*D*···*A*	*D*—H···*A*
N2—H2···N1^i^	0.89 (3)	2.27 (3)	3.142 (2)	168 (2)

Symmetry codes: (i) −*x*+1, −*y*+1, −*z*+1.
